# The Development of Upper Limb Movements: From Fetal to Post-Natal Life

**DOI:** 10.1371/journal.pone.0080876

**Published:** 2013-12-04

**Authors:** Stefania Zoia, Laura Blason, Giuseppina D’Ottavio, Marina Biancotto, Maria Bulgheroni, Umberto Castiello

**Affiliations:** 1 Department of Pediatrics, Institute for Maternal and Child Health IRCCS “Burlo Garofolo”, Trieste, Italy; 2 Department of Obstetrics and Gynecology, Institute for Maternal and Child Health IRCCS “Burlo Garofolo”, Trieste, Italy; 3 Ab.Acus, Biomedical Company, Milano, Italia; 4 Department of General Psychology, Faculty of Psychology, University of Padua, Padua, Italy; University of Bologna, Italy

## Abstract

**Background:**

The aim of this longitudinal study was to investigate how the kinematic organization of upper limb movements changes from fetal to post-natal life. By means of off-line kinematical techniques we compared the kinematics of hand-to-mouth and hand-to-eye movements, in the same individuals, during prenatal life and early postnatal life, as well as the kinematics of hand-to-mouth and reaching-toward-object movements in the later age periods.

**Methodology/Principal Findings:**

Movements recorded at the 14^th^, 18^th^ and 22^nd^ week of gestation were compared with similar movements recorded in an ecological context at 1, 2, 3, 4, 8, and 12 months after birth. The results indicate a similar kinematic organization depending on movement type (i.e., eye, mouth) for the infants at one month and for the fetuses at 22 weeks of gestation. At two and three months such differential motor planning depending on target is lost and no statistical differences emerge. Hand to eye movements were no longer observed after the fourth month of life, therefore we compared kinematics for hand to mouth with hand to object movements. Results of these analyses revealed differences in the performance of hand to mouth and reaching to object movements in the length of the deceleration phase of the movement, depending on target.

**Conclusion/Significance:**

Data are discussed in terms of how the passage from intrauterine to extra-uterine environments modifies motor planning. These results provide novel evidence of how different types of upper extremity movements, those directed towards one’s own face and those directed to external objects, develop.

## Introduction

In human development, hand-mouth contact is among the earliest cases of a sustained behavioral pattern that integrates two separate motor systems. The transport of hand(s) toward the mouth is manifested prenatally [1], and remains a prominent behavior after birth [2]. It is a hallmark of infancy, forming a basic act with obvious adaptive value through the lifespan. Hand-mouth behavior in newborns provides evidence that elements of coordination exist between the mouth and the hand at birth [3]. Such a notion is in contrast with earlier accounts that considered hand-mouth contacts at birth as merely fortuitous [4].

The expression of hand-mouth coordination develops rapidly during the months following birth. Morphological changes in hand-mouth coordination occur between 2 and 5 months, as infants start to transport objects to the mouth. Based on observations reported in the literature, the early development of hand-mouth coordination is shown to be closely related to the development of other sensory motor systems, in particular eye-hand coordination (e.g., [5]). For instance, assessing the development of hand-mouth coordination after the newborn period investigates the changes observed between 2–5 months in the coordination of reaching and grasping [6]. In these terms, hand-mouth integration changes developmentally, coming under the control of perceptual systems by 5 months of age when infants start to reach for [7]–[10], then to grasp [11], and finally to bring the objects to the mouth [12], [13].

The transitional phases between the early behavior that is independent of eye-hand coordination and later performances that integrate reaching and grasping have not yet been fully identified or analyzed in any systematic fashion. In the present longitudinal study, we explored this transition by investigating the differences between hand-to-mouth movements during prenatal life and early postnatal life, and between hand-to-mouth and reaching-toward-object movements in the later age periods.

Here, we capitalized on two studies that investigated the kinematic pattern of prenatal hand movements towards the mouth and the eye, establishing whether fetuses’ hand movements show some evidence of action planning depending on end goal [14], [15]. Zoia and colleagues [14] studied hand movements towards the mouth and towards the eye in fetuses aged 14, 18 and 22 weeks of gestation, showing that these hand movements are not casual. By means of off-line kinematical techniques it was demonstrated that the hand-to-mouth and the hand-to-eye movements are planned according to the size and/or delicacy of the target, suggesting a primitive predictive process in which the sensory consequences of a movement are anticipated and used for action planning. With respect to 14 and 18 weeks of gestation, at 22 weeks of gestation the peak velocity for movements towards the eye is reached earlier and is lower than that for movements towards the mouth, with a velocity profile characterized by a number of movement units that noticeably decreases. For movements towards the mouth duration was shorter, whereas the anticipation of peak velocity for movements directed towards the eye resulted in a longer deceleration time. This indicates that the hand movements of fetuses become directly aimed towards the target and that movement duration and velocity patterning may be elaborated in terms of the somatosensory properties of the target.

This apparent adaptation of the dynamics of hand movements to the target in fetuses contrasts with the slow improvement in the dynamics of similar movements during the first year and how these movements differ. Thus, the important question, which remains unanswered, concerns the characterization of the transitional changes in movement parameterization from the fetal to the neonatal periods. A longitudinal study comparing fetal and infant actions in analogous conditions is called for. Therefore, the main goal of this article is a kinematic investigation of the ontogenesis of actions directed towards one’s self and outwards, within the same group of participants before and after birth.

## Materials and Methods

### Ethics Statement

The experimental procedures were approved by the Institutional Review Board of the Institute for Maternal and Child Health – IRCCS “Burlo Garofolo”, and were in accordance with the Declaration of Helsinki (Sixth revision, 2008). All participants gave their informed written consent to participate in the study. The children’s legal guardians have viewed this full manuscript and have given written informed consent to publication of the photographs of their children, as outlined in the PLOS consent form.

### Participants

Eight healthy babies (2 females and 6 males) were studied from the 14^th^ week of gestation (GE) through the first year of life. During pregnancy, each of the eight fetuses was studied longitudinally at the 14^th^, 18^th^ and 22^nd^ week of gestation by undergoing a 20-minute four-dimensional-ultrasound (4D-US) observation session each time. Expecting mothers (aged 27 to 39 yrs) with a singleton pregnancy, attending the Institute for Maternal and Child Health – IRCCS “Burlo Garofolo”, were involved as a convenient sample of low-risk pregnant women. The designation of “low risk” for the fetus was determined following the first visit with an obstetrician on the basis of the mother’s medical history, and subsequently confirmed. All babies were full-term (natural childbirth between 38–41 GE), with a normal APGAR (range 9–10), normal birth-weight (range 3100–3900 g) and negative neurobehavioral assessment, performed one week after birth with the Brazelton Neonatal Behavioral Assessment Scale [16]. After birth, each baby was observed every 30 days between the first and the fourth month of life. Two subsequent visits were made at the 8^th^ and 12^th^ month of the babies’ age. At each visit infants were video-recorded for a 20-minute session. The experimental procedures were approved by the Institutional Review Board of the Institute for Maternal and Child Health – IRCCS “Burlo Garofolo”, and were in accordance with the Declaration of Helsinki (Sixth revision, 2008). All mothers gave written informed consent and approval at their first appointment.

### Procedure

During the prenatal period each woman was seen at the 14^th^, 18^th^, and 22^nd^ week of gestation. Each participating mother was identified by the prenatal ultrasound technician during her first visit at 12 weeks of pregnancy and fetal age was calculated comparing the mother’s last menstruation date and the measurements of the fetus (Crown Rump Length), as resulting from the ultrasound examination. To better visualize fetal movements the transducer was maintained stationary and positioned so that a frontal view of the fetus, including head, arms, hands, thorax and abdomen, was obtained. Each fetus was filmed for 20 minutes and no external stimulations were used to provoke fetal movements. However, each ultrasound video-recording was performed two hours after the mother’s lunch, in order to assure higher probability of an active waking. Following birth, each baby was visited at home at regular monthly intervals until the 4^th^ month of life and then again during the 8^th^ and the 12^th^ month of life. During the video recording performed at home, the position of each infant was standardized as follows: up to the 4^th^ month, all infants were recorded while seated in the same portable pram chair (with an inclination of 30 degrees up to the age of 3 months, and an inclination of 45 degrees subsequently). At 8 and 12 months, each infant was seated in the same high chair with an upright backrest. Babies’ upper limb movements were video-recorded during active wake. As before, no external stimulation was used to provoke upper limb movements until the age of 4 months, when babies were prompted to reach for different types of objects (a plastic cup or a ball) placed on the tray of the high chair, in the midline position, at a distance of 15 cm from the child.

### Instrumentation

Video-recording of prenatal upper limb movements was done by applying the abdominal four dimensional ultrasound technique (3D images in time, known as 4D-US; Voluson 730 Expert by GE Medical Systems). The ultrasound technique permits the variation of several parameters: the depth of the visual field, the sweeping angle that defines the sample volume and the frame rate. These parameters have a direct relationship to each other. In this study the machine was set at the fixed frame rate of 4 Hz, to guarantee the same number of images per sec. The crystal array of the transducer swept mechanically over the volume of the uterine cavity, framing the defined regions of interest (ROI). The video recordings were then digitized through our purposely developed software used for the off-line kinematic analysis of hand movements. Similarly, after birth, the upper limb movements were video-recorded with a sample frequency of 25 Hz per frame. The video camera was positioned in front of the baby. The films obtained were subsequently imported in an mpeg format and processed using our specific in-home made software, which permits to select, isolate and analyze each single upper limb movement. This is an off-line procedure that permits to obtain the desired kinematic parameters. Although a three-dimensional motion analysis system could have been used, we preferred to maintain similar data analysis for movements recorded during the prenatal and the postnatal periods, for better comparisons. Furthermore, some infants may not tolerate markers placed on their hands. This might impede an ecological means of data collection.

### Type of Movements

During the prenatal period two types of arm movements were isolated and evaluated by three experts and were subsequently analyzed: (i) hand to mouth, when hand movements end with finger to mouth contact and (ii) hand to eye, when hand movements end with finger to eye contact.

**Figure 1:**
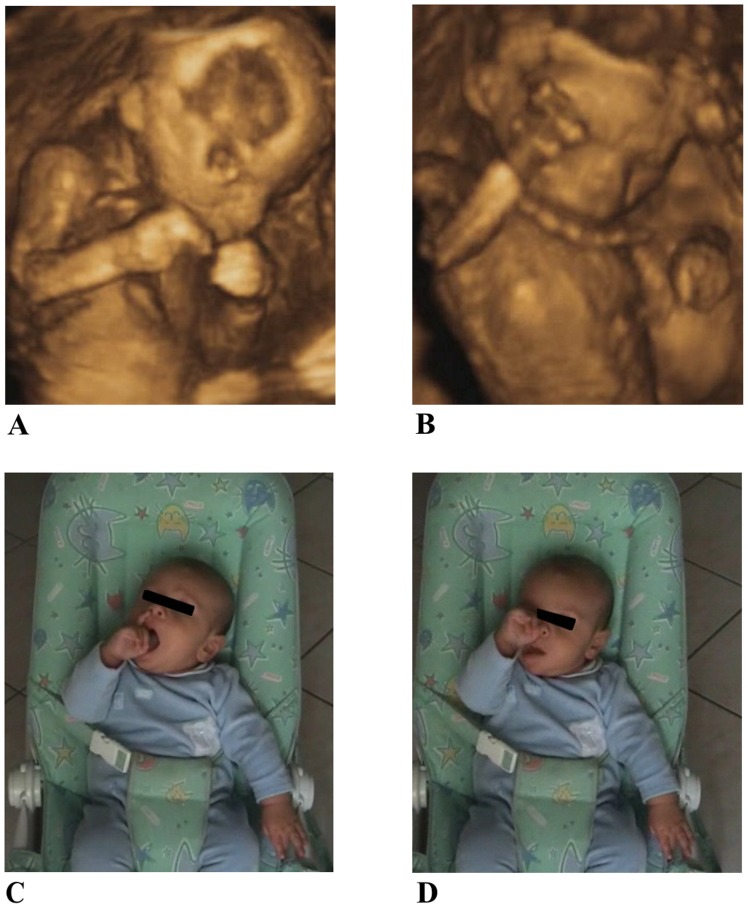
The two types of movements considered during the pre-natal period and the first 3 months of post-natal life: hand to mouth (A and C) and hand to eye (B and D) performed by a fetus at 18 weeks of gestation and by a 3 month old infant.

**Figure 2:**
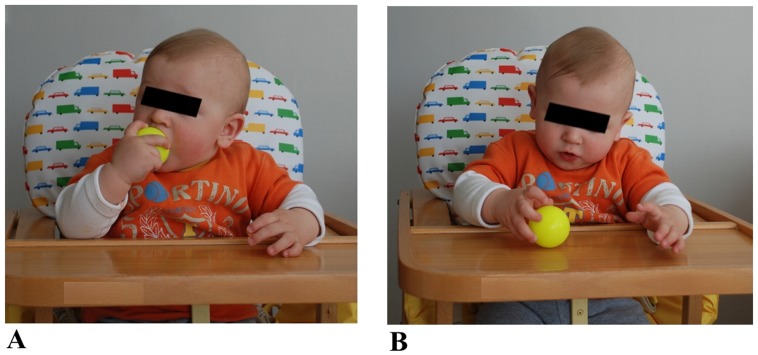
The two types of movements considered after the 4^th^ month of life: hand to mouth (A) and hand to object (B) performed by an 8 month old child.

Any of these movements were discarded from analysis if one of the following conditions occurred: the fetus was not in a supine position or he/she was not clearly visible from the starting to the end point or if the head was rotated so that the eye position was not in view. Criterion to determine the beginning of hand movements was when the hand was stationary within the chest area (below the shoulders and above the belly). The criteria for ‘touched target’ was when the hand clearly stopped on the mouth or the eye, for two consecutive frames. We took great care to discern target touch from proximity. Velocity change from zero was the threshold criterion for determining the start and end of the movement. Although many movements were detected, only those that met the above criteria were chosen for analysis within the 14^th^–22^nd^ week gestational period. During the first 3 months of postnatal life, similar types of arm movements were isolated and evaluated by three experts and subsequently analyzed: (i) hand to mouth, when hand movements ended with contact of finger to the mouth, (ii) hand to eye, when movements ended with contact of fingers to the eye (see Figure 1). After the 4^th^ month of life, hand-to-eye movements were no longer observed, therefore hand to mouth was compared with hand to object movements. Such movements ended with contact of fingers to the object (see Figure 2). For each participant, movement was analyzed by the same operator at all gestational ages and for all six postnatal sessions. To compare prenatal with postnatal data, similar criteria were adopted and movements were discarded from analysis if one of the following conditions occurred: hand movements started from non-visible positions or did not start in front of the thorax area (which was delimited vertically by both the shoulders line and the navel and horizontally by the maximum width of the shoulders) and/or the infant’s shoulders were not directly in front of the video-camera but diversely oriented. On the basis of these criteria only 60% of the observed movements were analyzed. Their frequency of occurrence is reported in Table 1.

**Table 1:** Frequency of occurrence for the three different types of movements analysed at three different times during the prenatal life and six different times during the first year of life for each foetus/infant.

Foetuses/Infants	14^th^ wk.	18^th^ wk.	22^th^ wk.	1^st^ mo.	2^nd^ mo.	3^rd^ mo.	4^th^ mo.	8^th^ mo.	12^th^ mo.
*Hand to eye*									
A.C.	3	2	1	1	1	0			
F.F.	2	2	1	1	0	1			
G.B	1	1	2	0	0	0			
G.R.	2	2	1	1	1	1			
L.S.	3	2	1	1	1	0			
M.V.	4	2	1	1	0	0			
B.A.	2	3	2	1	1	1			
P.D.	3	2	2	1	1	0			
*Hand to mouth*									
A.C.	2	2	2	1	2	2	5	8	6
F.F.	9	1	1	1	2	2	4	10	4
G.B	1	2	1	1	3	1	6	8	5
G.R.	1	1	2	0	1	3	2	12	7
L.S.	3	3	1	1	1	3	3	8	4
M.V.	4	4	2	1	2	1	6	6	5
B.A.	4	2	1	1	1	2	3	11	8
P.D.	4	3	2	1	3	2	6	7	9
*Hand to object*									
A.C.							3	5	6
F.F.							4	3	5
G.B							3	6	4
G.R.							4	5	7
L.S.							5	2	8
M.V.							3	8	4
B.A.							4	7	9
P.D.							4	5	7

*Notes:* wk = weeks; mo = months.

### Off-line Kinematic Analysis

This technique presents substantial difficulties. First of all, an absolute reference system cannot be established during the prenatal period (one of the times considered) therefore, to compare fetal and infant movements, a “baby-centered” coordinate system is needed. Secondly, the anthropometric parameters change from one fetus or infant to another, as well as with growth and age, therefore it was necessary to adopt relative measurements expressed in terms of inter-ocular distance for the fetuses (measured on the US images) and the length of the humerus bone for infants. This signifies that for each fetus and infant an absolute unit of measurement could not be applied. Thirdly, an off-line kinematic analysis can only be based on digital images that provide 2D coordinates, without the dimension of depth. For this purpose, a software application, specifically targeted to extract velocity and movement time measurements, was implemented. More precisely, the procedure consists in importing the ultrasound recordings of fetal movements and the videos recorded at home of the infants’ movements into the computer system. When the recordings are in the computer system, the specific single movements need to be identified and isolated. Then the relative reference system is selected and each movement can be marked by manually assigning a marker to the left shoulder, the right shoulder, the left eye and the right eye, the elbow, the wrist (and the fingers) and the entire movement has then to be tracked frame by frame, with respect to the target zone (eye, mouth and object). The wrist marker was used to compute arm velocity (displacement derivative) data. Marking was performed according to the same rules for all experimental video recordings: shoulder markers were identified in correspondence of the acromion position; the elbow marker was identified in correspondence of the olecranon protuberance; the wrist marker was identified by the ulna-styloid process; fingers were identified by the proximal phalanx of the index and eye markers were identified in correspondence of the eye caruncles. Because of the limited image quality of the US recordings and the presence of clothes on infants, case by case, the operator identified other landmarks, such as the local edge between the body and the background for US images or specific elements of the infants’ clothes to determine the positioning of the marker, frame by frame.

This procedure was performed manually and post-hoc by the same analyst for all fetuses. The coherence of the analysis between two independent experts was verified by using a specific type of intra-class correlation (all ICCs>.99). The following dependent measures were calculated and analyzed: (i) total movement time (MT, defined as the time at which the wrist first began to move until speed is close to zero, in milliseconds); (ii) the deceleration time in relative terms as a percentage of movement time (%DT, defined as the time spent from the peak velocity to the end of movement in relation to the total movement time) for the velocity profile.

## Data Analysis

For the dependent measures considered, two repeated analysis of variance (ANOVA) were conducted. In the first analysis two within-subjects factors were considered: type of movement (eye and mouth) and age period (14, 18, 22 weeks of gestation; 1, 2, 3 months after birth). For the second analysis, the two within-subjects factors were also type of movement, but mouth versus object, in addition to age period. Absolute temporal values were expressed in relative terms as a percentage of movement duration. Post-hoc contrasts were carried out with Bonferroni’s corrections for multiple comparisons.

## Results

### Kinematics of the Hand Trajectory

**Figure 3:**
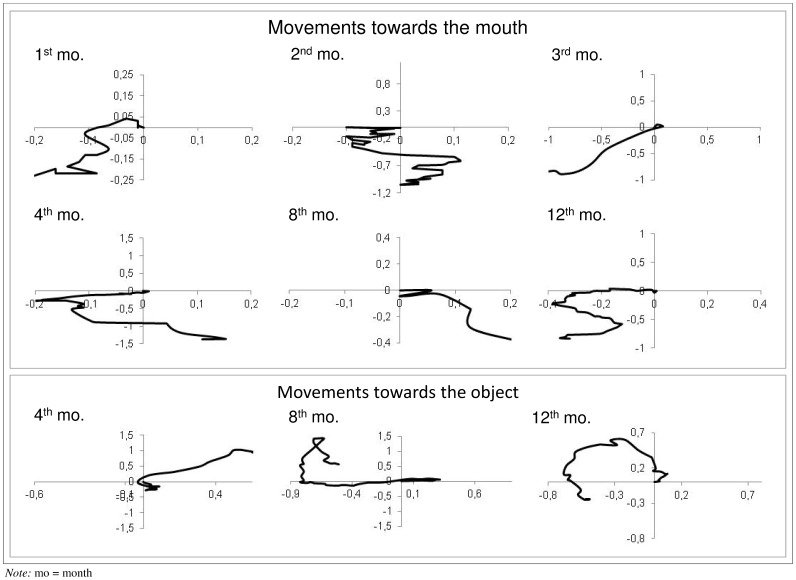
Examples of hand paths during movements towards the mouth and the object. The upper panel illustrates movements towards the mouth performed by the same infant at 1, 2, 3, 4, 8 and 12 months of age. The lower panel represents reaching movements towards the object at 4, 8 and 12 months of age by the same child. The origin of the referring system (0,0) indicates the position of the target. Measurement unit for both X and Y displacements is the length of the humerus bone.

Figure 3 shows the hand paths of movements to the mouth and to the object. The upper panels indicate the movements to the mouth of one infant at various ages (1, 2, 3, 4, 8, 12 months). It can be noticed that at older ages trajectory for movements to the mouth become longer, more complex and better controlled. The lower panels show the reaches towards objects of one infant at 4, 8 and 12 months of age. It can be noticed how the adjustments occurring during the last part of the trajectory change with age, showing a significant reduction at 12 months.

### Fetal versus Neonatal Upper Limb Movements: Comparing Movements Towards the Eye and the Mouth

**Figure 4:**
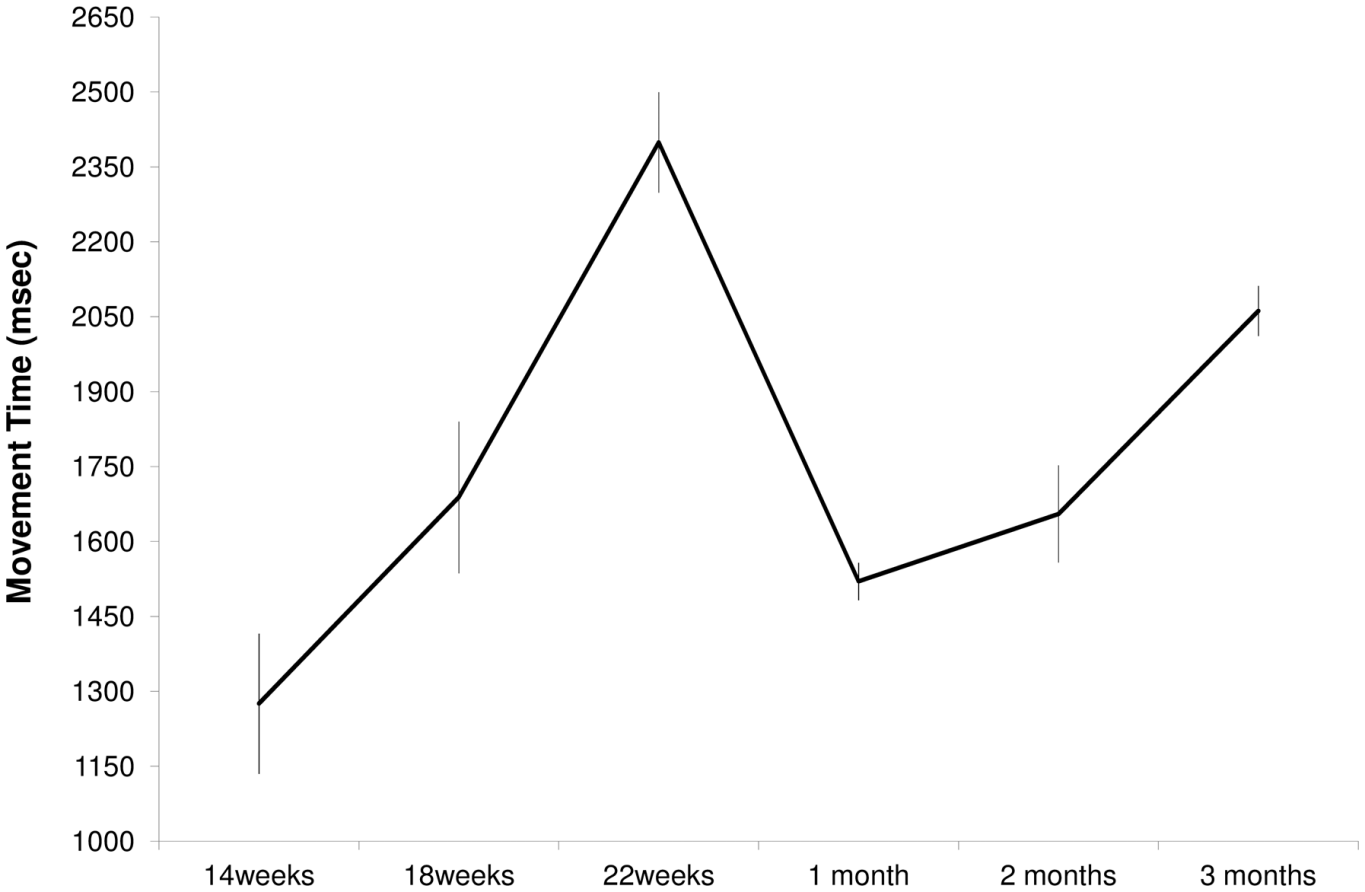
Movement Time, expressed in milliseconds, in the pre and post-natal periods.

For MT the ANOVA revealed a main effect of age (F_(5,3)_ = 36,53, p = 0.007, see Figure 4). Post-hoc contrasts revealed that MT was longer at 22 weeks compared to 14 (p = 0.001) and 18 weeks (p = 0.004) of gestation. Following birth, MT at one month was shorter than at 22 weeks of gestation (p = 0.001), two months (p = 0.001) and three months (p = 0.014) of postnatal life. The main effect of type of movement was also significant (F_(1,7)_ = 5,86, p = 0.046). Independent of gestational and age periods, hand movements to the eye were longer than those to the mouth (1893±60 vs. 1640±74 ms).

**Figure 5:**
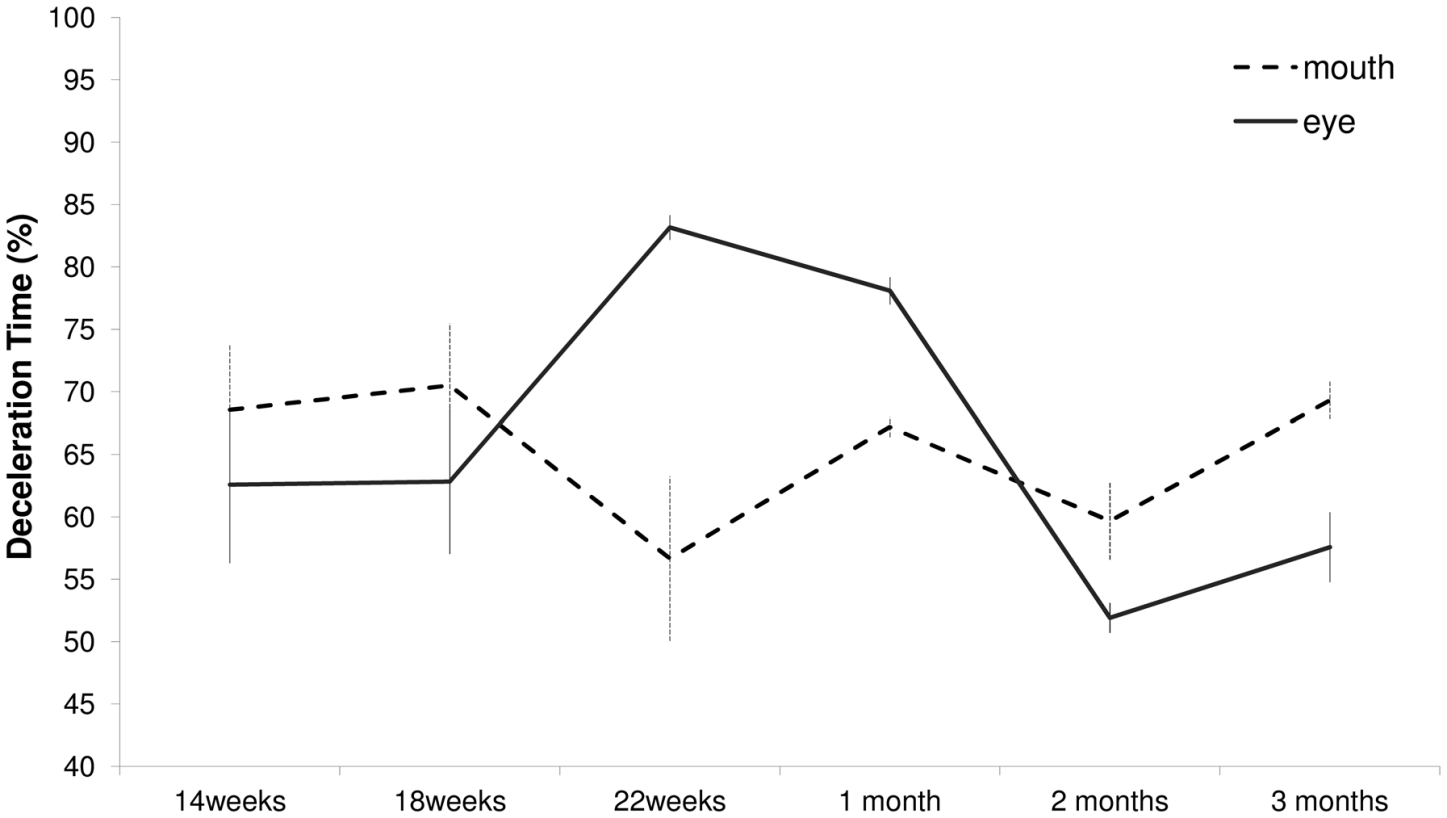
Deceleration time in percentage for movements performed towards the eye and towards the mouth at six different times before and after birth.

The ANOVA performed on %DT revealed a main effect of age (F_(5,3)_ = 11,82, p = 0.034). At 2 months of life %DT start to decelerate later than at 22 weeks of gestation (56% vs 70%; p = 0.006), at one (66%; p = 0.001) and three months (63%; p = 0.002) of postnatal life. In relative terms, %DT was shorter (i.e., later deceleration) at three months than at one month after birth (63% vs 66%; p = 0.001). Furthermore, a significant interaction between age periods and types of movement was also found (F_(5,3)_ = 204.64, p = 0.001). The results of this interaction are shown in Figure 5 and can be summarized as follows: at 22 weeks of gestation the percentage of time spent during deceleration was greater for arm movements performed towards the eye than towards the mouth (p = 0.006). This effect persists during the first month of life (p = 0.001), whereas at two and three months following birth deceleration time for movements performed towards the eye was shorter than those towards the mouth (p = 0.012 and p = 0.007, respectively). Thus, it is only at 22 weeks of gestation and at 1 month that the infants decelerate earlier for the eyes than for the mouth.

### Self versus Outer Upper Limb Movements

**Figure 6:**
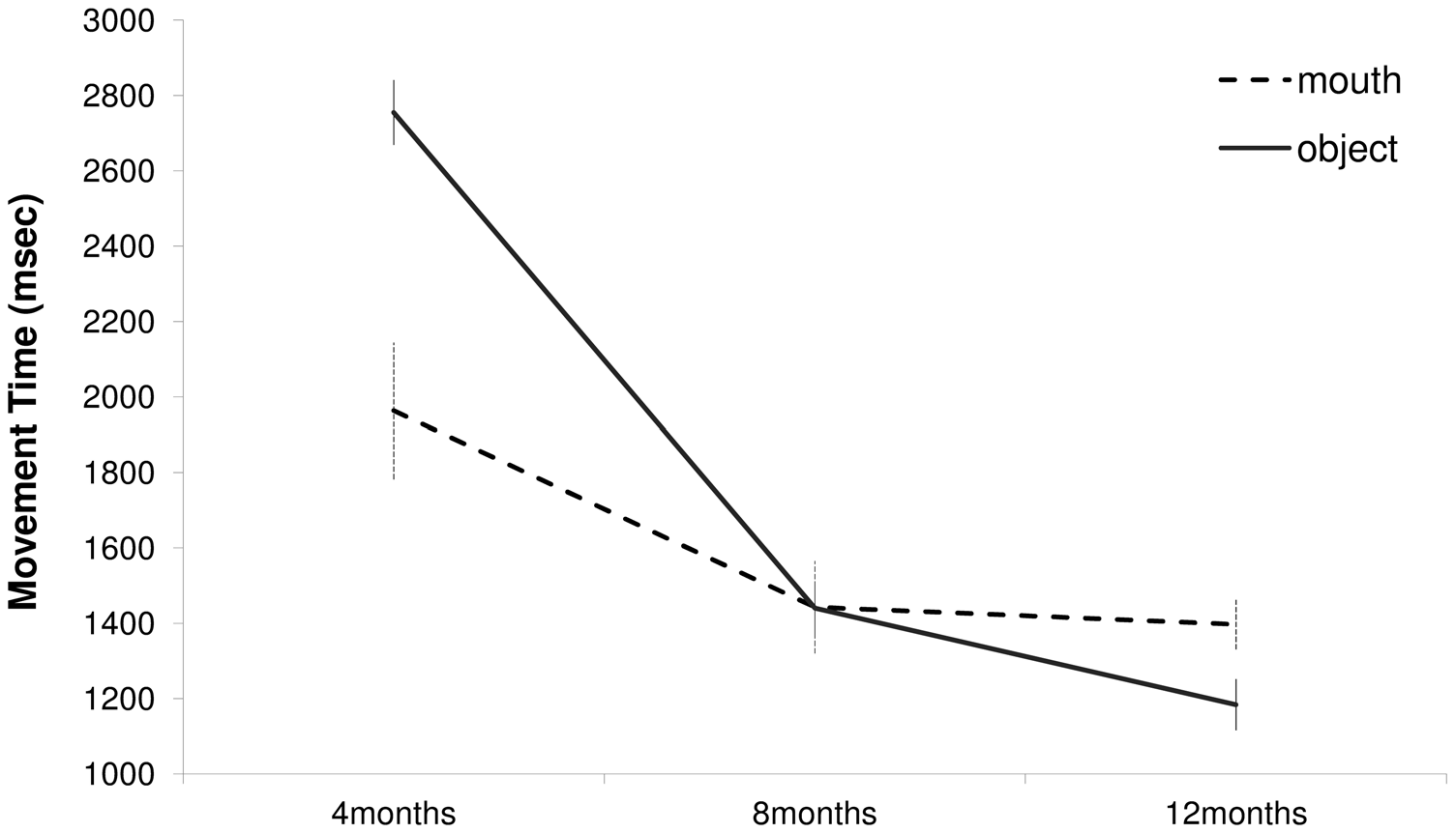
Movement Time for reaching to the mouth and to the object respectively at 4, 8 and 12 months of age.

In order to verify the influence of both experience and growth on the kinematic properties for upper limb movements, an ANOVA with age period (4, 8, 12 months) and type of movement (mouth, object) was carried out. The main effect of age period was significant (F_(2,6)_ = 93.19, p<0.001). MT was significantly shorter at 8 than 4 months (p<0.01), whereas no differences were found between 8 and 12 months. In addition, an interaction effect (age period × type of movement) was found (F_(2,6)_ = 7.22, p<0.025, see Figure 6): at 4 months MT for upper limb movements towards the object (2755 ms) was longer than that toward the mouth (1963 ms; p<0.016). On the contrary, at 12 months MT was shorter for the object (1184 ms) than for the mouth (1397 ms; p<0.009). At 8 months MT was not different for the two targets. Differences were also evident when considering deceleration time in relative terms. For this parameter a main effect of age period was found (F_(2,6)_ = 7.39, p<0.024). Deceleration time was longer at 12 (57%) compared to 8 (51%; p<0.005) and 4 (51% p<0.01) months. Furthermore, a main effect of type of movement was revealed (F_(1,7)_ = 34.84, p<0.001). Deceleration time was longer for movements towards the mouth (59%) compared to movements towards the object (47%). For this parameter no interaction effect was noticed.

## Discussion

The aim of this longitudinal study was to investigate how upper limb movements develop before and after birth. The present findings confirm that the temporal characteristics of fetal movements are by no means uncoordinated or without pattern. As previously reported, by 22 weeks of gestation the movements seem to show a recognizable form of motor planning, with kinematic patterns that depend on the target [14], [15]. Therefore, during development, the fetus would acquire motor skills, which reflect an “environment specific” maturation, similar to that shown during post-natal development in terms of pre- and reaching phases [17], [18]. This differential kinematic patterning suggests some motor behavior development, an increased level of motor control and some level of action planning in the fetus.

An important aspect of these results is that after birth the above mentioned kinematic patterning does not persist. This is witnessed by the fact that the longer movement duration and deceleration time found at 22 weeks of gestation for movements towards the eye rather than the mouth, re-emerge at four months of postnatal life. This suggests a possible reorganization phase, which might be necessary to re-adapt movement to the novel environment. Of course, a period of time might be needed to overcome the chaos possibly caused by two important post-natal changes, that is, acting in a richer and more challenging spatial context than in the uterus and the emergence of vision. Infants need to recalibrate actions within a non viscous and unlimited environment. In other words, there might be an environment specific maturation process that briefly persists after birth, but cannot be maintained when the infant becomes more aware of environmental changes.

At this stage, it appears important to understand which kind of calibration processes are taking place during the first three months of life. It might well be that following the first month of life the loss of planning ability is a result of infants’ active exploration of their own motor abilities and task constraints. Whether motor learning is described as soft assembling [19] or a search for task space [20], the present data show that it is after the first month of postnatal life that selective effective strategies for movement are explored. In other words, the development of hand movements might be the result of interactive learning to discover useful patterns of reaching which account for perceptual-motor interactions [21]. Whereas the role of proprioception might be essential in determining arm posture before birth, vision is a novel source of information for perceiving arm posture and the position of objects in the environment after birth. As our data indicate, assembling these two types of information may be a learning process that requires a few months.

In this regard, it could be of interest to compare the pattern of results obtained in the present study for movements directed outwards with those previously reported regarding the changes observed between 1–5 months in the coordination of reaching and grasping [8], [22]. This pattern of development has been explained in various manners. One view emphasizes the time needed for the development of eye-hand coordination [4], [23]. Another view considers the maturation of neuromuscular systems controlling arm movements as a prerequisite to successful reaching and grasping [22], [19]. These explanations might have different implications for the present results in terms of the relationship between self directed versus outward upper limb movements. Before entering this discussion, however, it is important to mention that after birth we found very few hand to eye movements, whereas hand to mouth persisted. This result is in line with the observation that, at 4 months of age, contacts on other parts of the face diminished, highlighting a focus on the mouth [6]. This suggests that after birth infants abandon movements of slamming into the eye, reason for which only the comparison between hand-to-mouth movements and reaching have been considered.

Hand to mouth coordination and reaching and grasping both have a directed arm movement component in common. These movements differ in that, for movements towards self, the target is mainly determined proprioceptively, whereas in reaching it is visually determined. If the decline in visually triggered directed reaching during the first three months of life is due to the maturation of the neuromuscular control of arm movements, similar changes should occur in hand-mouth and reaching-toward-object movements. If the decline is due to changes in eye-hand coordination, no such similarity should be evident, given that vision can neither specify nor completely guide the hand to target during hand to mouth coordination. Our precise measurements in an ecological setting show different characteristics for these two types of movement depending on age period. At 4 months, movement time for reaching towards the object was longer than that towards the mouth. At 8 months, movement time was not different for the two targets. At 12 months, MT was shorter for the object than for the mouth. These differences in movement time during the maturational period might indicate that by 12 months of age infants optimize motor planning on the basis of movement duration. This type of programming keeps the timing of the commands independent from the spatial parameters of movements. In other words, the selection of the muscles needed to be activated for a given task can be modified, or the torsion applied to the joints can be modulated within a centrally generated temporal pattern that determines the co-ordination of a given action. This might be the easiest and most readily chosen organizational option of the neural system during the maturation of neuromuscular control of arm movements to compensate for the postural and joint kinematic instability characterizing infants’ reaching actions. Indeed, the control of reaching movements takes a long time to be refined: kinematic studies have shown that jerky and zigzag movements represent the norm during the first month. Such movements are characterized by several movement units, typified by multiple accelerations and decelerations ([7]; see also [24], [25]). With growth, jerky movements progressively disappear in favor of a single movement unit that occupies a larger proportion of the reaching action [26].

Differences were also evident when considering the time spent during the deceleration phase. Deceleration time was longer for movements towards the mouth compared to movements towards the object, independent of age period. Movements towards the mouth could be considered a kind of investigative process for the oral exploration of objects, preceding a phase characterized by a more careful approach.

This study presents some limitations. The first is that it utilized two- rather than three-dimensional kinematics, but a two-dimensional approach was the only way to analyze fetal movements and infants movements in totally ecological, unconstrained conditions. The second is that because of technical restrictions, kinematical characterization was confined to a few dependent measures. Therefore a full fledged kinematic assessment was not possible.

In conclusion, despite the difficulties encountered in conducting this research project, these findings provide new information on the transitional changes in arm movements from the fetal to the neonatal periods. Our results support the idea that birth adaptation to a rich and challenging environment determines a reset of motor functions, which entails a de-novo motor learning for the re-establishment of distinct and recognizable kinematic patterns depending on end goal.
